# Electrocardiographic Predictors for Early Risk Stratification: 30-Day Mortality in Older Adult Trauma Patients

**DOI:** 10.3390/jcm14186659

**Published:** 2025-09-22

**Authors:** Sedat Ozdemir, Mehmet Murat Oktay, Iffet Tiftikci, Kazim Ersin Altinsoy

**Affiliations:** 1 Department of Internal Medicine, School of Medicine, Gaziantep Islam Science and Technology University, 27010 Gaziantep, Turkey; 2 Department of Emergency Medicine, School of Medicine, Gaziantep Islam Science and Technology University, 27010 Gaziantep, Turkey; mmurat_ok@yahoo.com (M.M.O.); ersinaltinsoy@gmail.com (K.E.A.); 3 Department of Emergency Medicine, School of Medicine, Gaziantep City Hospital, 27470 Gaziantep, Turkey; iffettiftikci@gmail.com

**Keywords:** electrocardiography, atrial fibrillation, older trauma, fracture, mortality, diagnostic, risk stratification

## Abstract

**Objectives:** This prospective observational study aimed to assess the prognostic value of electrocardiographic (ECG) findings obtained at emergency department (ED) admission in adult patients presenting with trauma-related fractures, with a focus on their association with 30-day all-cause mortality. **Materials and Methods:** A total of 391 patients aged ≥18 years with trauma-induced fractures were enrolled at a tertiary emergency center between February and May 2025. Baseline demographic and clinical data, including comorbidities, trauma mechanisms, and 12-lead ECG findings at admission, were recorded. Patients were monitored for 30-day mortality. Logistic regression analysis was used to identify independent predictors of mortality. **Results:** The mean age of the patients was 73.9 ± 6.7 years, and 50.1% were female. Normal sinus rhythm was the most common ECG finding (31.5%), followed by sinus tachycardia (20.5%) and bundle branch block (15.3%), while atrial fibrillation (AF) was present in 9.5% of cases. Thirty-day mortality occurred in 5.1% of the cohort (n = 20). Non-survivors had significantly higher frequencies of AF (35.0% vs. 8.1%, *p* = 0.001), head trauma (70.0% vs. 18.1%, *p* < 0.001), cerebrovascular disease (55.0% vs. 16.4%, *p* < 0.001), and polypharmacy (100% vs. 62.8%, *p* = 0.001) compared with survivors. Conversely, low-energy falls were more common among survivors (74.7% vs. 20.0%, *p* < 0.001), whereas falls from stairs or a bed and high-energy trauma were significantly more frequent among non-survivors (all *p* < 0.05). In multivariate logistic regression, AF (OR: 6.112; 95% CI: 1.612–23.176; *p* = 0.008), head trauma (OR: 16.514; 95% CI: 4.925–55.367; *p* < 0.001), and cerebrovascular disease (OR: 6.725; 95% CI: 2.219–20.385; *p* = 0.001) emerged as independent predictors of 30-day mortality. Although normal sinus rhythm was associated with survival in univariate analysis (*p* = 0.034), it did not retain independent significance in multivariate modeling. Patients with AF had significantly lower 30-day survival compared with those without AF (65.0% vs. 96.3%, *p* = 0.001). **Conclusions:** This prospective study demonstrates that electrocardiographic abnormalities—especially atrial fibrillation—are strong predictors of 30-day mortality in older adult trauma patients. Their prognostic value was further reinforced when assessed alongside head trauma and cerebrovascular disease. These findings emphasize ECG as a rapid, practical, and noninvasive tool for early risk stratification and clinical decision-making in the emergency care of geriatric fracture patients.

## 1. Introduction

Falls are one of the leading causes of traumatic injuries among adults and constitute a significant portion of emergency department (ED) admissions, especially in aging populations [1]. These injuries often result in fractures, which, although primarily orthopedic in nature, are frequently intertwined with underlying systemic conditions, particularly cardiovascular disorders [2,3]. The pathophysiological stress triggered by trauma can unmask or exacerbate cardiovascular comorbidities, rendering patients more vulnerable to complications such as arrhythmias and hemodynamic instability. Globally, falls represent the second leading cause of unintentional injury deaths, with more than 684,000 deaths annually, predominantly in individuals over 60 years of age. In the emergency department, up to 40% of adult trauma admissions are related to falls, most commonly resulting in fractures.

In this context, the electrocardiogram (ECG) serves not only as a routine diagnostic tool but also as a valuable modality for early risk assessment. Trauma-related autonomic activation, catecholamine surge, and inflammatory responses can lead to a variety of ECG abnormalities, including sinus tachycardia, bundle branch blocks, and, most notably, atrial fibrillation (AF) [4]. The pathophysiological interplay between trauma and ECG abnormalities is complex. Acute trauma triggers sympathetic activation, catecholamine surge, and systemic inflammation, all of which can precipitate arrhythmias. Furthermore, trauma-induced neurological dysfunction, including traumatic brain injury, may alter central autonomic regulation, leading to secondary electrocardiographic manifestations such as atrial fibrillation, conduction disturbances, and ST-T changes [5,6]. AF has been increasingly recognized as a significant contributor to early morbidity and mortality following trauma [7,8,9]. Moreover, pre-existing conditions such as hypertension, cerebrovascular disease, and previous intracranial pathology may further predispose individuals to post-traumatic arrhythmias [3,10].

Despite these associations, the prognostic value of ECG findings in trauma patients remains a subject of debate. Lenstra et al. reported that although ECG abnormalities are frequently observed in patients with traumatic brain injury, they may not independently predict in-hospital mortality [11]. This highlights the complexity of interpreting ECG findings in the trauma setting and underscores the necessity of evaluating them in conjunction with clinical context [12,13].

There is a relative paucity of prospective data examining the distribution and prognostic impact of ECG findings in adult patients presenting to the ED with trauma-related fractures [14,15]. In particular, the extent to which specific ECG abnormalities predict 30-day mortality remains inadequately defined. Identifying such relationships could offer critical insights for early risk stratification, especially in older adults or comorbid patients.

Despite these associations, there is still limited prospective evidence clarifying the prognostic role of electrocardiographic findings in older adult trauma patients. Recent studies have highlighted the link between trauma, autonomic dysregulation, and new-onset atrial fibrillation, which in turn is strongly associated with adverse outcomes and mortality in older adults [16,17,18]. Furthermore, data from neurocritical care settings suggest that early cardiac evaluation, including ECG, may provide crucial prognostic information beyond neurological assessment [19]. However, to date, no prospective study has systematically assessed the prognostic value of ECG findings specifically in older adult trauma patients with fractures. Addressing this gap may help establish ECG as an accessible, rapid, and noninvasive tool for early risk stratification in this vulnerable population.

Although electrocardiography is routinely used in trauma evaluation, its diagnostic and prognostic significance in older adult patients with fractures remains insufficiently defined. Therefore, the present study aimed to prospectively evaluate the prognostic value of ECG findings obtained at ED admission in adult trauma patients with fractures. Specifically, we sought to determine their association with 30-day mortality, while also assessing the impact of clinical comorbidities, trauma mechanisms, and localization. Clarifying these associations may facilitate early risk stratification and improve acute care decision-making in this vulnerable patient population.

## 2. Materials and Methods

### 2.1. Study Design

This study was designed as a prospective, descriptive, and observational investigation. It was conducted at the Emergency Department of Gaziantep City Hospital between 15 February and 31 May 2025. Prior to initiation, ethical approval was obtained from the Non-Interventional Clinical Research Ethics Committee of Gaziantep Islamic Science and Technology University (Protocol number: 2025-2ÖNP-0035). Written informed consent was obtained from all participants or their legally authorized representatives in accordance with the Declaration of Helsinki.

The study included adult patients aged 18 years and older who presented to the emergency department with trauma-related fractures. A 12-lead electrocardiogram (ECG) was recorded for each patient at the time of admission. Exclusion criteria were: (i) patients requiring immediate resuscitation, (ii) incomplete or missing clinical data, and (iii) lack of informed consent (or inability to obtain consent from legal representatives). A total of 391 patients who met the eligibility criteria were enrolled.

**Figure 1:**
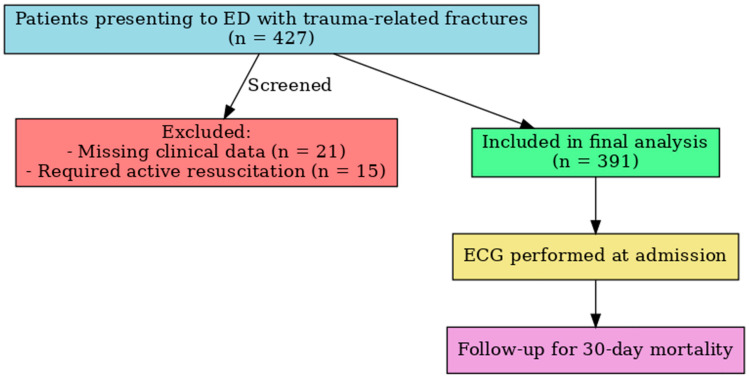
Flowchart of the study. ED = Emergency Department; ICU = Intensive Care Unit; ECG = Electrocardiogram.

Demographic data (age, sex), medical history (including diabetes mellitus, hypertension, cerebrovascular disease, coronary artery disease, and chronic kidney disease), trauma characteristics (mechanism and localization), and initial vital signs (heart rate, systolic and diastolic blood pressure, and peripheral oxygen saturation) were recorded using a standardized data collection form. Polypharmacy was defined as the concurrent use of five or more regular medications at the time of ED admission. The mechanisms of injury were categorized as low-energy fall, fall from stairs, fall from a bed, or high-energy fall. Trauma localization was classified by anatomical region, including head, thorax, pelvis, upper and lower extremities, and spinal segments. Head trauma was defined as clinically or radiologically confirmed injury involving the skull and/or brain (including skull fractures or traumatic brain injury), not limited to superficial scalp or soft-tissue injuries. Each patient was monitored throughout hospitalization, and 30-day mortality was recorded as the primary outcome. A total of 15 patients was excluded because they required immediate resuscitation, and an additional 21 patients were excluded due to incomplete data. Excluded patients were slightly younger and more often male compared with included patients, consistent with a higher proportion of high-energy trauma. For the final cohort, variable-level missingness was low (<5% for all variables). We used a complete-case analysis approach, with no imputation performed, as the small amount of missing data was unlikely to materially affect results (Figure 1).

### 2.2. ECG Evaluation

Twelve-lead ECGs obtained at admission were interpreted by the investigators. AF was defined as arrhythmia documented on the admission 12-lead ECG. Patients with a prior history of AF were identified from medical records and anticoagulant use; AF first detected at ED presentation was classified as new-onset AF. Findings were categorized as normal sinus rhythm (NSR), atrial fibrillation (AF), sinus tachycardia, sinus bradycardia, bundle branch block, extrasystole, T-wave inversion, first-degree atrioventricular (AV) block, pacemaker rhythm, or sinus arrhythmia. ECG categories were defined according to standard diagnostic criteria (e.g., NSR = 60–100 bpm with normal P-QRS-T sequence; AF = absence of P waves with irregularly irregular R-R intervals; tachycardia => 100 bpm; bradycardia =< 60 bpm). ECG tracings were independently reviewed by two investigators. Discrepancies were resolved by consensus, and a third reviewer was available in case of persistent disagreement. Inter-rater reliability between the two investigators was high, with a κ (kappa) coefficient of 0.87, corresponding to 93% agreement.

### 2.3. Statistical Analysis

All statistical analyses were performed using IBM SPSS Statistics version 27.0 (Armonk, NY, USA). No formal a priori power calculation was performed; however, the sample size (n = 391) was considered adequate for exploratory analysis. This sample size was consistent with previous prospective trauma cohorts of similar scope and was expected to provide sufficient precision for estimating effect sizes, even beyond the ≥10 events-per-variable guideline. Categorical variables were expressed as counts and percentages, while continuous variables were presented as mean ± standard deviation (SD), median, and interquartile range (IQR). The Kolmogorov–Smirnov test was used to assess the normality of distribution for continuous variables. Comparisons between groups were conducted using the Chi-square test for categorical variables and the Mann–Whitney U test for continuous variables without normal distribution. To evaluate predictors of 30-day mortality, we prespecified three core variables based on clinical relevance and univariate significance: atrial fibrillation, head trauma, and cerebrovascular disease. These analyses followed a prespecified statistical analysis plan (SAP), developed before examining outcome data, to minimize bias and ensure reproducibility. In addition, a minimal sufficient a priori adjustment set was defined comprising age, sex, heart rate at triage, trauma mechanism/severity (low-energy vs. stairs/bed/high-energy), and polypharmacy, which were forced into sensitivity models irrespective of univariate *p*-values. Multivariable modeling was performed using Firth penalized logistic regression to reduce small-sample bias. Sensitivity analyses included (i) a Firth penalized model additionally adjusting for the a priori confounders, and (ii) a ridge-penalized logistic regression including core predictors plus confounders. Exploratory analyses also included models adjusting for trauma mechanism categories (low-energy fall, stair fall, bed fall, high-energy trauma) and testing an interaction term between atrial fibrillation and head trauma (AF × head trauma) using Firth penalized logistic regression. Internal validation was conducted with bootstrap resampling (1000 iterations). Model performance was assessed by discrimination [area under the ROC curve (AUC) with 95% CI] and calibration (calibration plot and Brier score). A *p*-value < 0.05 was considered statistically significant.

## 3. Results

A total of 391 adult patients with trauma-related fractures were included in the study. The mean age of the cohort was 73.9 ± 6.7 years, and 50.1% of the participants were female, indicating an evenly distributed sex profile. Polypharmacy, a well-recognized marker of frailty in geriatric populations, was present in 64.7% of patients. Among the comorbidities, hypertension was the most prevalent (56.0%), followed by diabetes mellitus (34.5%), coronary artery disease (19.9%), and cerebrovascular disease (18.4%) (Table 1).

Electrocardiographic evaluation at the time of ED admission revealed normal sinus rhythm in 31.5% of patients. Abnormal ECG findings were common, with sinus tachycardia present in 20.5%, bundle branch block in 15.3%, and atrial fibrillation (AF)—a known marker of hemodynamic instability—in 9.5% of cases. Among the 37 patients with AF, a small subset had a known history of chronic AF, while the remainder represented new-onset AF at ED presentation. In a sensitivity analysis excluding chronic AF, the association between AF and 30-day mortality persisted, suggesting that incident AF largely drove the observed risk. Regarding trauma localization, head trauma emerged as the most frequent site of injury (20.7%), followed by injuries involving the upper (22.0%) and lower extremities (15.6%). The predominant mechanism of injury was low-energy fall, accounting for 71.9% of all cases. The extent of missingness across clinical and ECG variables was low (<5%), and complete-case analysis was used without imputation (Table 1).

Among the 391 patients included in the study, 20 individuals (5.1%) died within 30 days. Of these, 14 deaths occurred in hospital and 6 occurred post-discharge. The leading causes were intracranial complications of head trauma, cardiovascular events (including AF-related stroke and heart failure), and multi-organ failure after high-energy trauma. No cases were attributable to withdrawal of care. When baseline characteristics were compared between survivors and non-survivors, several significant differences emerged. Polypharmacy was universally present among non-survivors (100%) compared to 62.8% of survivors (*p* = 0.001), suggesting a strong association between multiple medication use and short-term mortality. Hypertension (80.0% vs. 54.7%, *p* = 0.026) and coronary artery disease (40.0% vs. 18.9%, *p* = 0.021) were also significantly more prevalent in the non-survivor group. Notably, cerebrovascular disease was found in 55.0% of non-survivors versus only 16.4% of survivors (*p* < 0.001).

In contrast, the prevalence of diabetes mellitus and chronic kidney disease did not differ significantly between groups. Vital sign measurements, including systolic and diastolic blood pressure, oxygen saturation, and age, were similar across both groups, with no statistically significant differences. However, heart rate demonstrated a non-significant trend toward being higher in non-survivors (94.6 vs. 88.2 bpm, *p* = 0.055) (Table 2).

Comparison of ECG findings and trauma characteristics between survivors and non-survivors revealed several clinically and statistically significant differences. Atrial fibrillation (AF) was significantly more frequent among patients who died within 30 days (35.0% vs. 8.1%, *p* = 0.001), identifying it as a strong predictor of poor short-term outcomes. Conversely, normal sinus rhythm was more common in survivors (32.6% vs. 10.0%, *p* = 0.034), although this association did not persist in multivariate analysis. Trauma localization also differed significantly between groups. Head trauma was markedly more prevalent among non-survivors (70.0% vs. 18.1%, *p* < 0.001), while lower extremity injuries were more frequent in survivors (16.4% vs. 0.0%, *p* = 0.048). Regarding trauma mechanism, low-energy falls were significantly associated with survival (74.7% vs. 20.0%, *p* < 0.001), whereas falls from stairs (40.0% vs. 14.3%, *p* = 0.002) or a bed (20.0% vs. 7.3%, *p* = 0.040) and high-energy falls (20.0% vs. 3.8%, *p* = 0.001) were disproportionately more common among patients who died (Table 3).

**Figure 2:**
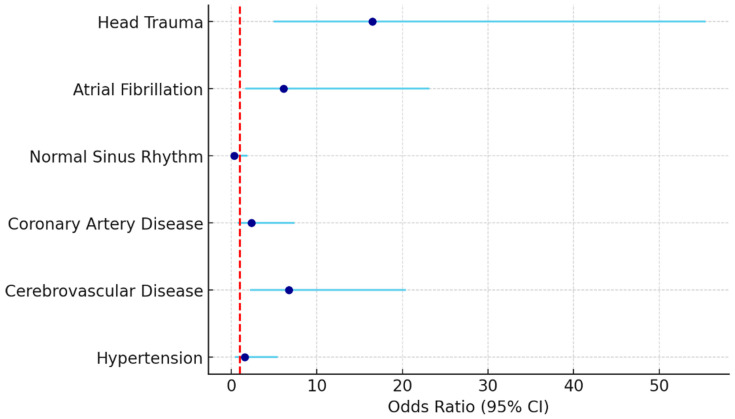
Forest plot of multivariate logistic regression for 30-day mortality.

Multivariate logistic regression analysis identified three independent predictors of 30-day mortality in patients with trauma-related fractures. The strongest association was observed with head trauma, which increased the risk of death more than sixteenfold (OR: 16.514, 95% CI: 4.925–55.367, *p* < 0.001). Cerebrovascular disease also emerged as a significant risk factor, with an odds ratio of 6.725 (95% CI: 2.219–20.385, *p* = 0.001), highlighting the vulnerability of patients with pre-existing neurologic conditions. Furthermore, atrial fibrillation remained an independent predictor of mortality (OR: 6.112, 95% CI: 1.612–23.176, *p* = 0.008), reinforcing its clinical significance even after adjustment for comorbidities and trauma characteristics. Other variables such as hypertension, coronary artery disease, and normal sinus rhythm were not independently associated with mortality in the adjusted model (*p* > 0.05 for all) (Table 4, Figure 2).

**Figure 3:**
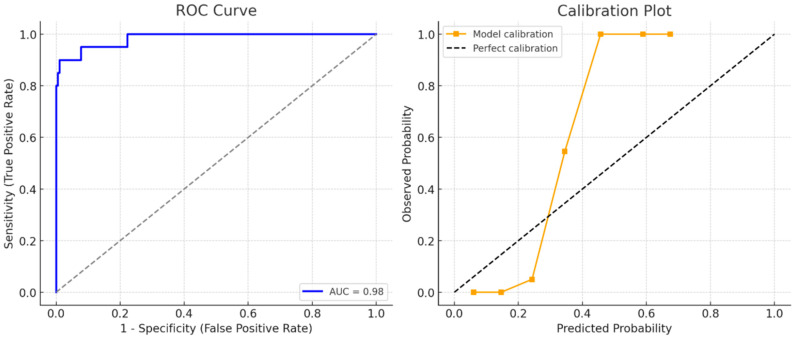
ROC Curve and Calibration Plot of the Penalized Logistic Regression Model for 30-Day Mortality.

ROC Curve and Calibration Plot of the Penalized Logistic Regression Model for 30-Day Mortality are shown in Figure 3. In Firth penalized regression, atrial fibrillation (OR: 5.4, 95% CI: 1.6–17.9), head trauma (OR: 14.8, 95% CI: 4.3–51.2), and cerebrovascular disease (OR: 5.9, 95% CI: 1.9–18.7) remained significant independent predictors of 30-day mortality. The optimism-corrected AUC of the model was 0.98, indicating excellent discrimination, while the Brier score was 0.06, reflecting very good calibration. The calibration plot demonstrated close agreement between predicted and observed probabilities (Figure 3). These findings highlight atrial fibrillation as a strong predictor of short-term mortality, with patients exhibiting AF showing significantly higher odds of 30-day mortality compared to those without AF (*p* = 0.006).

To address potential confounding, we performed a Firth penalized regression model additionally adjusting for age, sex, heart rate, trauma mechanism/severity, and polypharmacy. The associations for atrial fibrillation, head trauma, and cerebrovascular disease persisted, with modest attenuation of effect sizes. Results were consistent in a ridge-penalized logistic regression including the same covariates. When trauma mechanism categories (low-energy fall, stair fall, bed fall, high-energy trauma) were included in the penalized regression, head trauma remained a significant independent predictor of 30-day mortality, though the effect size was modestly attenuated. In addition, we tested for an interaction term between AF and head trauma (AF × head trauma), but this was not statistically significant (*p* > 0.10), suggesting additive rather than multiplicative effects. Model performance remained stable across these sensitivity analyses (optimism-corrected AUC ≈ 0.95; Brier score ≈ 0.07). A summary of model specifications and sensitivity analyses is presented in Supplementary Table S1.

## 4. Discussion

In this prospective study, we evaluated the prognostic significance of electrocardiographic (ECG) findings, comorbidities, trauma mechanisms, and injury localization in predicting 30-day mortality among adult patients presenting to the emergency department (ED) with trauma-related fractures. Our results demonstrated that atrial fibrillation (AF), head trauma, and a history of cerebrovascular disease were independently associated with short-term mortality, underscoring the utility of ECG as a rapid, noninvasive risk stratification tool in the acute trauma setting.

Among the ECG abnormalities, atrial fibrillation emerged as the strongest predictor of mortality. In our cohort, 35% of non-survivors had AF compared to only 8.1% of survivors (*p* = 0.001), and multivariate analysis confirmed its independent association with a sixfold increased risk of death. This finding was reinforced by sensitivity analysis, which indicated that new-onset AF was the main driver of early mortality, consistent with trauma-related autonomic and inflammatory stress. Chronic AF, although less common, also contributed to adverse outcomes, reflecting underlying cardiovascular vulnerability. Curfman et al. reported that AF significantly worsened outcomes in older adult trauma patients [7]. The underlying mechanisms likely involve trauma-induced sympathetic overdrive, systemic inflammation, and myocardial strain, which may precipitate new-onset AF in vulnerable individuals [17,20,21]. Moreover, AF itself has been implicated in increasing fall risk, suggesting a possible bidirectional relationship between trauma and arrhythmogenesis [22].

Other ECG abnormalities, such as bundle branch block, premature contractions, and bradycardia, were not independently linked to mortality in our study. However, these findings may still reflect increased age-related conduction disturbances or underlying cardiac disease, as suggested by Al-Khouja et al. [23], who emphasized the prognostic relevance of conduction abnormalities in trauma settings. Although normal sinus rhythm was associated with survival in univariate analysis (*p* = 0.034), this effect did not persist in multivariate modeling (*p* = 0.237), indicating that its apparent protective role may be mediated by other clinical variables.

Head trauma was a particularly critical determinant of adverse outcomes. It was present in 70% of non-survivors compared to only 18.1% of survivors (*p* < 0.001), and it remained the strongest independent predictor of mortality in our adjusted model (OR: 16.514). These results are consistent with prior literature highlighting the unique vulnerability of older adult patients to traumatic brain injury (TBI) [24]. In this population, even minor head trauma may result in severe consequences due to factors such as cerebral atrophy and frequent use of antithrombotic medications. Additionally, recent studies have proposed that head trauma may exert a neurocardiogenic influence on the heart through central autonomic dysregulation [25,26,27,28]. Stewart et al. [16] demonstrated a significant association between TBI and subsequent development of AF, suggesting a mechanistic link that may partially explain our findings. In our cohort, most deaths occurred in hospital and were primarily related to head trauma or cardiovascular complications, indicating that competing events such as withdrawal of care were not major contributors. Because head trauma often co-occurs with high-risk mechanisms such as stair or bed falls, part of its prognostic impact may reflect mechanism severity. However, in models adjusting for trauma mechanism, head trauma retained independent significance, supporting its role as a robust predictor of short-term mortality.

Polypharmacy, although not identified as an independent predictor in multivariate analysis, was present in all non-survivors, highlighting its potential role in both fall risk and adverse post-traumatic outcomes [29,30]. Medications affecting cardiovascular function, cognition, and balance (e.g., sedatives, antihypertensives, antipsychotics) may impair compensatory mechanisms and increase vulnerability to trauma [31,32]. The American Heart Association has acknowledged polypharmacy as a critical contributor to fall-related morbidity in older adults with cardiovascular disease [33].

Our analysis of trauma mechanisms also revealed insightful trends. Low-energy falls were the most common injury mechanism (71.9%) and were significantly more frequent among survivors (74.7% vs. 20.0%, *p* < 0.001). In contrast, falls from stairs, falls from a bed, and high-energy trauma were all significantly associated with mortality. These results are consistent with the findings of Zaskey et al., who reported that stair-related falls were associated with disproportionately higher morbidity and mortality, even in the absence of high-impact forces [34]. This suggests that the context and biomechanics of the fall, rather than just its energy level, play a crucial role in prognosis. Taken together, our findings provide compelling evidence that early ECG evaluation—particularly the detection of AF—alongside clinical assessment of head trauma and cerebrovascular disease, can enhance prognostic accuracy in older adult trauma patients. By applying penalized regression and bootstrap internal validation, we reduced the risk of model overfitting and confirmed the robustness of AF, head trauma, and cerebrovascular disease as predictors. The model demonstrated excellent discrimination (AUC 0.98) and very good calibration (Brier score 0.06), which aligns with prior studies reporting similar performance metrics in older adult trauma populations [7,9,16,17]. These parameters are easily obtainable upon ED admission and may inform timely clinical interventions, closer monitoring, or ICU triage. Importantly, we addressed potential overfitting by applying penalized logistic regression and bootstrap validation. This methodological refinement confirmed the robustness of AF, head trauma, and cerebrovascular disease as independent predictors, despite the limited number of events. The internal validation results (AUC and Brier score) support the reliability and generalizability of our prognostic model.

Our findings that AF, head trauma, and a history of cerebrovascular disease independently predict 30-day mortality in older adult trauma patients are biologically plausible. Acute trauma provokes a catecholamine surge, heightened sympathetic drive, and systemic inflammation, which together lower the threshold for atrial ectopy and facilitate re-entry, thereby triggering AF in susceptible atrial substrates [17,20,21]. Inflammation-related oxidative stress and calcium-handling abnormalities can further promote atrial remodeling and electrical instability, sustaining AF beyond the initial adrenergic trigger [18]. Consistent with these mechanisms, post-traumatic arrhythmias—including new-onset AF—are frequently observed in older adults with fractures and are associated with worse short-term outcomes [7,9,17].

Head trauma may amplify these processes through neurocardiogenic pathways. Traumatic brain injury can disrupt the central autonomic network (e.g., insular cortex and brainstem circuits), producing sympathetic–parasympathetic imbalance that manifests as ECG abnormalities—AF, conduction disturbances, and repolarization changes—even in the absence of primary cardiac pathology [11,12,13,25,26,27,28]. Recent cohort data suggest that TBI increases subsequent risk of AF/flutter, supporting a causal link between neurotrauma and arrhythmogenesis that aligns with our observation of head injury as the strongest predictor of mortality [16]. Beyond arrhythmia risk, head trauma in older adults often coexists with antithrombotic use and cerebral atrophy, raising the likelihood of secondary brain injury and hemodynamic instability, which may compound early mortality.

The independent association between cerebrovascular disease and death likely reflects reduced autonomic reserve, impaired baroreflex function, and coexisting small-vessel pathology that heighten vulnerability to both neurogenic and systemic stressors after trauma [18,27]. Polypharmacy—universal among non-survivors in our cohort—can exacerbate this risk via QT-prolonging or negatively inotropic agents and drug–drug interactions that degrade cardiovascular and cognitive compensatory mechanisms during the acute phase [29,30,31,32]. Finally, the distribution of injury mechanisms (e.g., stair or bed falls) plausibly marks higher odds of head impact and rapid shifts in intracranial and autonomic tone, helping to explain their disproportionate association with mortality compared with low-energy ground-level falls [34].

Clinically, these mechanistic considerations justify early ECG-based triage coupled with vigilant neurologic assessment in older adult trauma patients. Detecting AF at admission should prompt closer monitoring (e.g., telemetry), correction of reversible precipitants (pain, hypoxia, electrolyte imbalance), and early involvement of cardiology, particularly when head injury or cerebrovascular comorbidity coexists. Integrating ECG findings with trauma characteristics into ED pathways may improve disposition decisions (ward vs. ICU) and enable targeted prevention of early adverse events.

This study possesses several noteworthy strengths that enhance the reliability and clinical relevance of its findings. Foremost among them is its prospective design, which allowed for systematic and real-time collection of clinical and electrocardiographic data. By assessing ECG findings at the point of admission and following patients for 30-day outcomes, we were able to evaluate temporal relationships between early physiological disturbances and short-term mortality. This design minimizes recall bias and strengthens the causal inferences that can be drawn from our analyses. Another key strength lies in the focus on accessible and low-cost diagnostic parameters, such as the 12-lead ECG. The identification of atrial fibrillation as a strong and independent predictor of mortality reinforces the practical value of ECG as a rapid, noninvasive risk stratification tool that can be implemented in virtually all emergency settings, including resource-limited environments. Furthermore, by incorporating variables such as polypharmacy, comorbid conditions, trauma mechanism, and head injury, we provided a comprehensive clinical framework for interpreting ECG findings in the context of broader physiological and functional vulnerability. In addition, the study cohort consisted predominantly of older adult patients, a population that is frequently underrepresented in trauma research despite their disproportionately high rates of morbidity and mortality. Our findings therefore offer important insights for geriatric emergency care and may contribute to the development of risk assessment algorithms tailored to older adults.

### Limitations of the Study

This study has some limitations. First, although ECG interpretations were performed by trained physicians, inter-observer variability was not formally assessed. Future studies incorporating blinded, independent ECG analysis or inter-rater reliability metrics would improve reproducibility. Second, the single-center nature of the study may limit the generalizability of our findings to other institutions with different patient populations, trauma epidemiology, or management protocols. In addition, the relatively short enrollment period (3.5 months) may not fully capture potential seasonal variation or referral patterns, further limiting transportability of the findings. Additionally, we did not include biochemical cardiac markers such as troponin, CK-MB, or BNP, which may have provided further prognostic context when evaluated alongside ECG findings. These markers could help differentiate between structural myocardial injury and functional arrhythmias, thereby refining risk stratification. Lastly, while we focused on 30-day all-cause mortality as a robust and clinically meaningful outcome, data on in-hospital complications, readmissions, or functional recovery were not captured. We also excluded 15 patients who required immediate resuscitation and 21 patients with incomplete data. This approach, while necessary to ensure consistency and reliability of ECG evaluation, may have introduced selection bias by removing the sickest cases and those with insufficient information. Consequently, our study may underestimate absolute mortality rates and attenuate the strength of associations between ECG abnormalities and outcomes. Therefore, our findings are most applicable to older adult trauma patients who are initially stable and for whom ECG assessment at admission is feasible. Despite these limitations, the study offers strong preliminary evidence supporting the early use of ECG and clinical profiling to identify high-risk patients with trauma-related fractures. Future multicenter studies with larger cohorts and expanded biomarker panels are warranted to validate and extend these findings.

## 5. Conclusions

In conclusion, we demonstrated that electrocardiographic findings—particularly atrial fibrillation—are strongly associated with 30-day mortality. Alongside AF, head trauma and a history of cerebrovascular disease emerged as independent predictors of poor outcomes. These results highlight the prognostic importance of early ECG evaluation in the emergency setting, not only as a diagnostic tool but also as a means of risk stratification in vulnerable trauma patients.

Given the high prevalence of comorbidities and polypharmacy in this population, our findings underscore the importance of incorporating ECG into the initial evaluation of older adult trauma patients. In practice, detecting atrial fibrillation at ED admission should alert clinicians to a significantly increased risk of short-term mortality. Such patients may benefit from closer monitoring, early cardiology consultation, and consideration for higher-level care (e.g., ICU admission). Likewise, identifying head trauma and pre-existing cerebrovascular disease at presentation may further refine early risk stratification and inform tailored management strategies.

Overall, ECG remains an accessible, low-cost, and clinically informative tool for emergency physicians. Its ability to rapidly identify patients at elevated risk makes it a valuable adjunct in triage and decision-making, particularly in resource-limited settings where advanced diagnostics may not be readily available. Future research should focus on multicenter validation studies with larger and more diverse populations, integration of cardiac biomarkers with ECG findings, and exploration of long-term outcomes including functional recovery, rehospitalization, and quality of life. Such efforts may refine prognostic models and foster the development of standardized risk stratification tools for older adult trauma patients.

## Figures and Tables

**Table 1:** Demographic, Clinical, and Trauma Characteristics of the Patients.

Characteristic	n (%) or Mean ± SD/Median (IQR)
Gender	
Male	195 (49.9%)
Female	196 (50.1%)
Polypharmacy	253 (64.7%)
Comorbidities	
Diabetes Mellitus	135 (34.5%)
Hypertension	219 (56.0%)
Cerebrovascular Disease	72 (18.4%)
Coronary Artery Disease	78 (19.9%)
Electrocardiographic Findings	
Normal Sinus Rhythm	123 (31.5%)
Bundle Branch Block	60 (15.3%)
T Wave Inversion	36 (9.2%)
First-degree AV Block	14 (3.6%)
Sinus Tachycardia	80 (20.5%)
Atrial Fibrillation	37 (9.5%)
Sinus Arrhythmia	15 (3.8%)
Pacemaker Rhythm	8 (2.0%)
Extrasystole	11 (2.8%)
Sinus Bradycardia	7 (1.8%)
Trauma Localization	
Head	81 (20.7%)
Thorax	45 (11.5%)
Upper Extremity	86 (22.0%)
Lower Extremity	61 (15.6%)
Pelvis	52 (13.3%)
Thoracic Spine	31 (7.9%)
Lumbar Spine	35 (9.0%)
ED Disposition	
Discharged	234 (59.8%)
Admitted to Ward	118 (30.2%)
Admitted to ICU	39 (10.0%)
Mechanism of Injury	
Low-energy fall	281 (71.9%)
Fall from stairs	61 (15.6%)
Fall from bed	31 (7.9%)
High-energy fall	18 (4.6%)
30-day Mortality	20 (5.1%)
Vital Signs	
Age (years)	73.9 ± 6.7/73 (11)
Systolic BP (mmHg)	128.9 ± 22.9/126 (32)
Diastolic BP (mmHg)	72.3 ± 15.3/69 (18)
Heart Rate (bpm)	88.5 ± 17.8/88 (22)
SpO_2_ (%)	95.3 ± 3.1/95 (4)

**Table 2:** Comparison of Demographic and Clinical Characteristics According to 30-day Mortality.

Characteristic	Survivors (n = 371)	Non-Survivors (n = 20)	*p*-Value †
	n (%) or Mean ± SD/Median (IQR)		
Gender			
Male	184 (49.6)	11 (55)	0.638
Female	187 (50.4)	9 (45)	
Polypharmacy	233 (62.8%)	20 (100%)	0.001 **
Diabetes Mellitus	127 (34.2%)	8 (40.0%)	0.597
Hypertension	203 (54.7%)	16 (80.0%)	0.026 *
Cerebrovascular Disease	61 (16.4%)	11 (55.0%)	<0.001 **
Coronary Artery Disease	70 (18.9%)	8 (40.0%)	0.021 *
Chronic Kidney Disease	49 (13.2%)	2 (10.0%)	0.678
Vital Signs			*p*-value ††
Age (years)	73.9 ± 6.8/73 (11)	73.9 ± 6.3/74 (13)	0.909
Systolic BP (mmHg)	128.8 ± 22.9/126 (32)	132.4 ± 23.3/127 (37)	0.522
Diastolic BP (mmHg)	72.0 ± 15.3/69 (18)	77.2 ± 15.5/72 (19)	0.177
Heart Rate	88.2 ± 17.9/87 (23)	94.6 ± 15.2/95 (25)	0.055
SpO_2_ (%)	95.3 ± 3.1/95 (4)	95.5 ± 3.3/96 (4)	0.627

Chi-square test (†) and Mann–Whitney U test (††) were used for comparisons. * *p* <0.05, ** *p* < 0.01 were considered statistically significant.

**Table 3:** Comparison of ECG Findings, Trauma Characteristics, and Clinical Outcomes According to 30-day Mortality.

	Survivors (n = 371)	Non-Survivors (n = 20)	*p*-Value
	N (%)		
Electrocardiographic Findings			
Normal Sinus Rhythm	121 (32.6)	2 (10)	0.034 *
Bundle Branch Block	58 (15.6)	2 (10)	0.496
T Wave Inversion	34 (9.2)	2 (10)	0.900
First-degree AV Block	13 (3.5)	1 (5)	0.726
Sinus Tachycardia	74 (19.9)	6 (30)	0.278
Atrial Fibrillation	30 (8.1)	7 (35)	0.001 **
Sinus Arrhythmia	15 (4)	-	0.359
Pacemaker Rhythm	8 (2.2)	-	0.507
Extrasystole	11 (3)	-	0.435
Sinus Bradycardia	7 (1.9)	-	0.535
Trauma Localization			
Head	67 (18.1)	14 (70)	<0.001 **
Thorax	45 (12.1)	-	0.098
Upper Extremity	84 (22.6)	2 (10)	0.184
Lower Extremity	61 (16.4)	-	0.048 *
Pelvis	50 (13.5)	2 (10)	0.656
Thoracic Spine	31 (8.4)	-	0.178
Lumbar Spine	33 (8.9)	2 (10)	0.866
Mechanism of Injury			
Low-energy fall	277 (74.7)	4 (20)	<0.001 **
Fall from stairs	53 (14.3)	8 (40)	0.002 **
Fall from bed	27 (7.3)	4 (20)	0.040 *
High-energy fall	14 (3.8)	4 (20)	0.001 **

Categorical variables were compared using the Chi-square test. * *p* < 0.05 was considered statistically significant; ** *p* < 0.01 was considered highly significant. Hyphen (-) indicates no patients in that subgroup.

**Table 4:** Multivariate Logistic Regression Analysis for Predictors of 30-day Mortality.

Variables	*p*-Value	OR	%95 CI	
			Lower	Upper
Hypertension	0.478	1.568	0.453	5.434
Cerebrovascular Disease	0.001	6.725	2.219	20.385
Coronary Artery Disease	0.141	2.359	0.752	7.406
Normal Sinus Rhythm	0.237	0.370	0.071	1.921
Atrial Fibrillation	0.008	6.112	1.612	23.176
Head Trauma	<0.001	16.514	4.925	55.367
Intercept	<0.001	0.115		

OR = Odds Ratio; CI = Confidence Interval.

## Data Availability

The raw data supporting the conclusions of this article will be made available by the corresponding author on request.

## Supplementary Materials

The following supporting information can be downloaded at: https://www.mdpi.com/article/10.3390/jcm14186659/s1. The STROBE checklist for cohort studies (downloadable from https://www.strobe-statement.org/checklists/ accessed on 27 August 2025) is included as Supplementary Material and will be published alongside the manuscript. Supplementary Table S1. Penalized Logistic Regression Models for 30-Day Mortality.
