# Supplementary material for: Electrocardiographic Predictors for Early Risk Stratification: 30-Day Mortality in Older Adult Trauma Patients
Source: J Clin Med. 2025 Sep 22;14(18):6659. doi: 10.3390/jcm14186659 (PMC12470298; doi:10.3390/jcm14186659)
Supplement: Supplementary file 1 [file jcm-14-06659-s001.zip › jcm-3874490-supplementary.pdf]

Supplementary Table S1. Penalized Logistic Regression Models for 30-Day Mortality.

Model	Predictors included	Penalization method	OR (95% CI) for AF	OR (95% CI) for Head trauma	OR (95% CI) for Cerebrovascular disease	AUC (95% CI)	Brier score	Notes
**Primary model**	AF, Head trauma, Cerebrovascular disease	Firth penalized logistic regression	5.4 (1.6–17.9)	14.8 (4.3–51.2)	5.9 (1.9–18.7)	0.98 (0.93–1.00)	0.06	Prespecified core predictors only
**Sensitivity model 1**	AF, Head trauma, Cerebrovascular disease + Age, Sex, HR, Mechanism/severity, Polypharmacy	Firth penalized logistic regression	4.9 (1.4–17.0)	13.1 (3.8–47.5)	5.2 (1.7–16.5)	0.95 (0.89–0.99)	0.07	Forced a priori confounders
**Sensitivity model 2**	AF, Head trauma, Cerebrovascular disease + Age, Sex, HR, Mechanism/severity, Polypharmacy	Ridge-penalized logistic regression	5.1 (1.5–16.9)	13.6 (4.1–45.9)	5.4 (1.8–16.9)	0.95 (0.88–0.99)	0.07	Penalized shrinkage for stability
